# Supplementary material for: A Likelihood Approach for Real-Time Calibration of Stochastic Compartmental Epidemic Models
Source: PLoS Comput Biol. 2017 Jan 17;13(1):e1005257. doi: 10.1371/journal.pcbi.1005257 (PMC5240920; doi:10.1371/journal.pcbi.1005257)
Supplement: S1 File — (TAR.GZ) [file pcbi.1005257.s014.tar.gz › HSPH_Online-SI-Revision/MSS10/n100-mild/n100-mild_NEW_table8.pdf]

1	R0	{1.24125, 3.38982, 0.1}	{1.22544, 3.07157, 0.}	{{1.22544, 3.07157, 0.}, {1.22544, 3.07157, 0.}}	{1.27396, 9.38834, 100.}	inf	161.	142.351	{92.8237, 186.551}	21.5234	0.111387	newly	287.	262.86	{155., 347.}	22.5226	0.114008	Reff	0.815836	0.805671	{0.80498, 0.806265}	1.24595	ar	34 926.	34 829.4	{34 472., 35 140.}	0.613526
2	R0	{1.29554, 4.51878, 0.1}	{1.31748, 4.25564, 2.}	{{1.31748, 4.25564, 2.}, {1.31748, 4.25564, 2.}}	{1.69333, 5.82329, 1900.}	inf	262.	319.221	{268.314, 366.394}	22.2207	0.0848068	newly	367.	431.9	{357., 505.}	19.4005	0.0752517	Reff	0.75267	0.764661	{0.764031, 0.765327}	1.59317	ar	42 828.	42 950.7	{42 720., 43 230.}	0.433105
3	R0	{1.2443, 3.64292, 0.1}	{1.25474, 3.26445, 0.}	{{1.25474, 3.26445, 0.}, {1.25474, 3.26445, 0.}}	{0.838956, 10.3892, 100.}	inf	255.	154.206	{104.874, 195.012}	39.6114	0.232209	newly	387.	265.56	{171., 344.}	31.9742	0.181315	Reff	0.790168	0.798062	{0.797482, 0.79865}	0.999011	ar	37 087.	36 971.5	{36 718., 37 218.}	0.519292
4	R0	{1.38465, 3.43331, 0.1}	{1.37974, 3.3318, 5.}	{{1.37974, 3.3318, 5.}, {1.37974, 3.3318, 5.}}	{0.354677, 2.95676, 4900.}	inf	64.	53.555	{30.0186, 77.9724}	26.5643	0.142743	newly	86.	83.2	{44., 121.}	29.814	0.142541	Reff	0.700356	0.698016	{0.697666, 0.698332}	0.3341	ar	49 511.	49 531.7	{49 443., 49 623.}	0.123286
5	R0	{1.25409, 4.1068, 0.1}	{1.25648, 3.67615, 2.19}	{{1.25474, 3.26445, 0.}, {1.25656, 3.82585, 3.}}	{0.190442, 10.4862, 2144.}	inf	297.	246.223	{192.678, 288.641}	17.6149	0.0895808	newly	386.	382.35	{304., 448.}	12.1425	0.0542776	Reff	0.780997	0.783122	{0.782211, 0.783549}	0.272099	ar	38 571.	38 525.2	{38 267., 38 827.}	0.464079
6	R0	{1.28821, 4.2016, 0.1}	{1.28596, 3.98513, 0.}	{{1.28596, 3.98513, 0.}, {1.28596, 3.98513, 0.}}	{0.174748, 5.15203, 100.}	inf	242.	230.789	{189.483, 279.527}	12.98	0.0590546	newly	315.	328.36	{263., 409.}	13.2825	0.0553274	Reff	0.761538	0.760352	{0.759639, 0.760876}	0.155817	ar	41 468.	41 570.7	{41 284., 41 885.}	0.432092
7	R0	{1.24369, 2.56007, 0.1}	{1.24751, 2.5364, 0.}	{{1.24751, 2.5364, 0.}, {1.24751, 2.5364, 0.}}	{0.307391, 0.924408, 100.}	inf	25.	28.7912	{5.39115, 56.7632}	65.0165	0.321213	newly	53.	57.74	{8., 121.}	61.5472	0.336213	Reff	0.764869	0.767173	{0.766783, 0.767465}	0.301207	ar	38 603.	38 592.6	{38 485., 38 754.}	0.221511
8	R0	{1.232, 3.53942, 0.1}	{1.22544, 3.07157, 0.}	{{1.22544, 3.07157, 0.}, {1.22544, 3.07157, 0.}}	{0.532715, 13.2182, 100.}	inf	101.	87.3186	{44.9719, 126.333}	27.9957	0.168295	newly	163.	159.2	{81., 237.}	28.8221	0.160417	Reff	0.809486	0.805341	{0.804845, 0.805845}	0.512003	ar	34 555.	34 623.9	{34 388., 34 896.}	0.477529
9	R0	{1.36262, 3.85631, 0.1}	{1.38889, 4.25615, 4.}	{{1.38889, 4.25615, 4.}, {1.38889, 4.25615, 4.}}	{1.92813, 10.3684, 3900.}	inf	139.	173.5	{129.342, 206.296}	26.8552	0.099855	newly	200.	228.94	{175., 275.}	19.79	0.0773563	Reff	0.708181	0.721356	{0.720861, 0.721912}	1.86046	ar	48 410.	48 485.5	{48 329., 48 661.}	0.241954
10	R0	{1.28702, 4.67968, 1.}	{1.28969, 4.0489, 0.56}	{{1.28596, 3.98513, 0.}, {1.29927, 4.21286, 2.}}	{0.325958, 13.4792, 100.}	inf	267.	228.73	{172.606, 293.296}	18.2739	0.091175	newly	358.	324.16	{239., 412.}	16.7542	0.0814443	Reff	0.767643	0.769727	{0.766768, 0.775473}	0.324302	ar	41 092.	41 055.7	{40 746., 41 395.}	0.466758
11	R0	{1.35752, 3.47129, 0.1}	{1.31535, 3.47086, 5.}	{{1.31535, 3.47086, 5.}, {1.31535, 3.47086, 5.}}	{3.10618, 0.0124187, 4900.}	inf	143.	154.575	{109.001, 194.934}	19.7626	0.0831927	newly	225.	240.17	{170., 308.}	20.6267	0.0880093	Reff	0.755256	0.731644	{0.731099, 0.732209}	3.12634	ar	44 669.	44 794.2	{44 568., 44 998.}	0.369787
12	R0	{1.35929, 5.83647, 0.1}	{1.34002, 5.27199, 5.}	{{1.34002, 5.27199, 5.}, {1.34002, 5.27199, 5.}}	{1.41758, 9.67161, 4900.}	inf	598.	504.965	{463.155, 548.614}	15.5578	0.0745371	newly	613.	566.47	{511., 619.}	8.59869	0.0399232	Reff	0.753917	0.744476	{0.743872, 0.745019}	1.25222	ar	46 715.	45 843.5	{45 583., 46 133.}	1.86555
13	R0	{1.28019, 3.29908, 0.1}	{1.29991, 3.11702, 3.}	{{1.30094, 3.11158, 3.}, {1.30094, 3.11158, 3.}}	{1.60425, 5.51836, 2900.}	inf	135.	105.684	{57.1535, 149.433}	26.8922	0.157456	newly	244.	183.85	{95., 262.}	29.5451	0.172877	Reff	0.759998	0.772086	{0.772075, 0.773301}	1.65389	ar	40 941.	40 957.1	{40 729., 41 165.}	0.346841
14	R0	{1.36661, 3.48318, 0.1}	{1.37819, 3.32361, 4.84}	{{1.37974, 3.3318, 5.}, {1.37974, 3.3318, 5.}}	{0.920494, 4.58122, 4740.}	inf	77.	74.3482	{42.411, 100.671}	23.9541	0.117328	newly	115.	117.76	{64., 163.}	26.9739	0.125612	Reff	0.705239	0.71125	{0.711416, 0.71245}	0.921849	ar	48 672.	48 578.4	{48 445., 48 712.}	0.236851
15	R0	{1.35865, 3.84123, 0.1}	{1.37916, 3.32872, 4.94}	{{1.37974, 3.3318, 5.}, {1.37974, 3.3318, 5.}}	{1.50938, 13.3422, 4840.}	inf	163.	104.59	{62.5993, 144.711}	36.0337	0.223904	newly	228.	164.97	{95., 233.}	29.8816	0.182148	Reff	0.704243	0.715678	{0.715363, 0.71655}	1.62376	ar	48 598.	48 364.	{48 205., 48 518.}	0.494362
16	R0	{1.22879, 3.98329, 0.1}	{1.2253, 3.0838, 0.05}	{{1.22544, 3.07157, 0.}, {1.22544, 3.07157, 0.}}	{0.284362, 22.5816, 146.}	inf	367.	277.932	{206.486, 345.62}	24.7058	0.130913	newly	573.	518.37	{393., 649.}	17.0838	0.082935	Reff	0.822699	0.821451	{0.820632, 0.822415}	0.152623	ar	34 405.	34 169.5	{33 659., 34 580.}	0.980642
17	R0	{1.34925, 3.64524, 0.1}	{1.36035, 3.22945, 3.}	{{1.36035, 3.22945, 3.}, {1.36035, 3.22945, 3.}}	{0.822573, 11.4063, 2900.}	inf	162.	108.735	{66.6937, 150.859}	33.2193	0.194406	newly	237.	176.1	{102., 250.}	28.5738	0.162634	Reff	0.721606	0.728266	{0.727664, 0.728821}	0.922986	ar	46 871.	46 744.1	{46 576., 46 901.}	0.332295
18	R0	{1.33054, 2.25275, 0.1}	{1.31246, 2.23442, 4.}	{{1.31246, 2.23442, 4.}, {1.31246, 2.23442, 4.}}	{1.35899, 0.813541, 3900.}	inf	9.	8.99045	{1.96256, 17.0685}	51.0074	0.270202	newly	21.	19.44	{2., 38.}	55.0476	0.338898	Reff	0.740885	0.730816	{0.73071, 0.730904}	1.35898	ar	44 336.	44 342.4	{44 310., 44 377.}	0.0517638
19	R0	{1.28394, 3.69859, 0.1}	{1.25753, 3.27768, 0.63}	{{1.25474, 3.26445, 0.}, {1.26656, 3.29313, 3.}}	{2.05976, 11.5353, 688.}	inf	204.	160.945	{112.032, 207.602}	23.2978	0.123909	newly	360.	270.04	{175., 349.}	25.8722	0.142299	Reff	0.786067	0.770441	{0.768225, 0.775894}	1.99164	ar	39 558.	39 271.3	{38 973., 39 491.}	0.761489
20	R0	{1.2883, 2.26493, 0.1}	{1.31081, 2.22167, 4.}	{{1.31246, 2.23442, 4.}, {1.31246, 2.23442, 4.}}	{1.85323, 1.90991, 3900.}	inf	22.	42.9682	{13.2834, 68.9959}	109.531	0.311394	newly	37.	93.99	{37., 162.}	161.865	0.385921	Reff	0.763434	0.776497	{0.777041, 0.77782}	1.81733	ar	40 768.	40 897.5	{40 772., 41 053.}	0.3268
21	R0	{1.34098, 2.78621, 0.1}	{1.31235, 2.2752, 4.05}	{{1.31246, 2.23442, 4.}, {1.31246, 2.23442, 4.}}	{2.13527, 19.2873, 3950.}	inf	39.	28.1024	{5.56777, 48.9471}	41.8493	0.2922	newly	64.	59.23	{12., 105.}	42.5156	0.254665	Reff	0.744378	0.728627	{0.728337, 0.728976}	2.11606	ar	44 605.	44 552.5	{44 465., 44 633.}	0.158615
22	R0	{1.33783, 5.95911, 0.1}	{1.34002, 5.27199, 5.}	{{1.34002, 5.27199, 5.}, {1.34002, 5.27199, 5.}}	{0.163768, 11.5306, 4900.}	inf	686.	613.233	{551.355, 665.762}	10.7612	0.0504137	newly	709.	696.	{631., 763.}	6.44852	0.0284485	Reff	0.760757	0.762978	{0.762119, 0.763724}	0.291943	ar	45 418.	44 932.1	{44 591., 45 267.}	1.08688
23	R0	{1.33359, 3.3069, 0.1}	{1.36035, 3.22945, 3.}	{{1.36035, 3.22945, 3.}, {1.36035, 3.22945, 3.}}	{2.00651, 2.342, 2900.}	inf	84.	84.0108	{46.4765, 117.613}	28.5287	0.13487	newly	143.	139.25	{76., 199.}	27.5035	0.13335	Reff	0.719592	0.73403	{0.733564, 0.734532}	2.00649	ar	46 245.	46 280.3	{46 107., 46 419.}	0.214899
24	R0	{1.30588, 2.56123, 0.1}	{1.31246, 2.23442, 4.}	{{1.31246, 2.23442, 4.}, {1.31246, 2.23442, 4.}}	{0.503724, 12.7598, 3900.}	inf	11.	13.0532	{2.86936, 25.5161}	59.341	0.258164	newly	29.	29.39	{6., 58.}	54.1724	0.284343	Reff	0.752997	0.756763	{0.756596, 0.756896}	0.500146	ar	42 383.	42 377.5	{42 334., 42 428.}	0.0808815
25	R0	{1.29813, 4.51849, 0.1}	{1.31602, 4.25222, 2.}	{{1.31748, 4.25564, 2.}, {1.31748, 4.25564, 2.}}	{1.37825, 5.89298, 1900.}	inf	473.	396.814	{339.893, 448.69}	16.2525	0.0794586	newly	633.	538.3	{464., 616.}	15.3302	0.0747711	Reff	0.756044	0.767466	{0.767238, 0.768928}	1.51082	ar	43 371.	42 928.1	{42 591., 43 240.}	1.05209
26	R0	{1.33231, 3.99953, 0.1}	{1.3172, 4.15362, 2.39}	{{1.31535, 3.47086, 2.}, {1.31748, 4.25564, 5.}}	{1.13401, 7.28939, 2290.}	inf	227.	226.466	{180.503, 267.125}	11.4714	0.0508544	newly	332.	306.83	{256., 359.}	11.991	0.0564007	Reff	0.747852	0.739378	{0.738787, 0.739901}	1.13308	ar	44 733.	44 506.9	{44 267., 44 745.}	0.560951
27	R0	{1.30555, 4.61445, 0.1}	{1.29927, 4.21286, 2.}	{{1.29927, 4.21286, 2.}, {1.29927, 4.21286, 2.}}	{0.480714, 8.70283, 1900.}	inf	364.	315.61	{265.345, 359.276}	14.2475	0.0691683	newly	467.	431.21	{359., 497.}	11.5824	0.0544207	Reff	0.764165	0.76112	{0.760498, 0.76176}	0.39844	ar	42 624.	42 396.7	{42 135., 42 665.}	0.593797
28	R0	{1.26819, 5.34969, 0.1}	{1.25646, 3.82822, 3.02}	{{1.25656, 3.82585, 3.}, {1.25656, 3.82585, 3.}}	{0.92469, 28.4403, 2920.}	inf	578.	369.441	{306.251, 427.23}	36.0829	0.198523	newly	683.	566.53	{452., 674.}	17.4978	0.087998	Reff	0.806442	0.801605	{0.800915, 0.802434}	0.599745	ar	38 946.	37 643.	{37 194., 38 036.}	3.34566
29	R0	{1.19925, 3.00394, 0.1}	{1.22019, 2.90246, 0.48}	{{1.19264, 2.01465, 0.}, {1.22544, 3.07157, 3.}}	{1.92243, 7.1605, 548.}	inf	160.	158.119	{94.7975, 217.749}	23.8427	0.116611	newly	328.	319.75	{202., 431.}	21.7957	0.106512	Reff	0.835841	0.850456	{0.831947, 0.854694}	1.90186	ar	31 727.	31 145.6	{30 629., 31 598.}	1.91937
30	R0	{1.35749, 3.75483, 0.1}	{1.36907, 3.27551, 3.9}	{{1.36035, 3.22945, 3.}, {1.37974, 3.3318, 5.}}	{0.853358, 12.7655, 3800.}	inf	77.	67.6476	{34.9035, 101.498}	26.4614	0.139182	newly	120.	106.81	{51., 154.}	27.725	0.145813	Reff	0.704592	0.710732	{0.705934, 0.716454}	0.871505	ar	48 244.	48 251.1	{48 122., 48 387.}	0.163564
31	R0	{1.27131, 4.2161, 0.1}	{1.25474, 3.26445, 0.}	{{1.25474, 3.26445, 0.}, {1.25474, 3.26445, 0.}}	{1.30345, 22.5719, 100.}	inf	338.	226.43	{172.41, 272.606}	33.066	0.182409	newly	482.	388.73	{288., 482.}	21.2303	0.109811	Reff	0.797836	0.788837	{0.788224, 0.7895}	1.12799	ar	38 299.	37 966.9	{37 590., 38 354.}	0.984282
32	R0	{1.31827, 6.18119, 0.1}	{1.34002, 5.27199, 5.}	{{1.34002, 5.27199, 5.}, {1.34002, 5.27199, 5.}}	{1.64996, 14.7092, 4900.}	inf	827.	586.624	{532.898, 635.819}	29.066	0.150116	newly	844.	670.71	{607., 716.}	20.532	0.100801	Reff	0.757649	0.773371	{0.772633, 0.774078}	2.0751	ar	45 262.	44 125.4	{43 676., 44 509.}	2.51107
33	R0	{1.20398, 1.35167, 0.1}	{1.2663, 1.35135, 3.}	{{1.2663, 1.35135, 3.}, {1.2663, 1.35135, 3.}}	{5.17577, 0.0236939, 2900.}	inf	3.	21.7191	{4.7661, 41.4932}	625.324	0.753275	newly	6.	76.05	{16., 152.}	1171.17	0.981462	Reff	0.802742	0.844053	{0.843783, 0.844264}	5.14624	ar	33 329.	33 448.1	{33 340., 33 617.}	0.357917
34	R0	{1.32776, 3.146, 0.1}	{1.31023, 3.04996, 5.}	{{1.31023, 3.04996, 5.}, {1.31023, 3.04996, 5.}}	{1.32062, 3.0527, 4900.}	inf	21.	27.9771	{7.21833, 49.0288}	63.1611	0.252493	newly	25.	46.11	{12., 84.}	104.28	0.32132	Reff	0.733149	0.723376	{0.723099, 0.723642}	1.33309	ar	44 872.	44 853.9	{44 782., 44 938.}	0.10784
35	R0	{1.279, 1.11336, 0.1}	{1.30222, 1.35944, 3.}	{{1.30222, 1.35944, 3.}, {1.30222, 1.35944, 3.}}	{1.81542, 22.1022, 2900.}	inf	0.1	5.66594	{2.06065, 9.9401}	5565.94	1.68304	newly	0.1	17.87	{4., 39.}	17 770.	2.11448	Reff	0.754188	0.767806	{0.76775, 0.767852}	1.80564	ar	41 033.	41 057.9	{41 037., 41 087.}	0.0606341
36	R0	{1.20029, 1.94717, 0.1}	{1.19264, 2.01465, 3.}	{{1.19264, 2.01465, 3.}, {1.19264, 2.01465, 3.}}	{0.637407, 3.46562, 2900.}	inf	45.	38.9485	{8.53267, 73.1021}	44.8727	0.281917	newly	117.	99.52	{23., 172.}	44.5983	0.27925	Reff	0.807351	0.802277	{0.801869, 0.802635}	0.628467	ar	33 234.	32 890.5	{32 734., 33 066.}	1.03551
37	R0	{1.33216, 3.69685, 0.1}	{1.3266, 3.41051, 4.5}	{{1.31535, 3.22945, 3.}, {1.36035, 3.47086, 5.}}	{1.47523, 7.74559, 4400.}	inf	150.	126.779	{81.6624, 160.687}	19.5229	0.103395	newly	219.	196.87	{122., 248.}	18.9635	0.0965319	Reff	0.735299	0.73254	{0.725882, 0.751417}	1.46285	ar	45 235.	45 114.5	{44 954., 45 264.}	0.318382
38	R0	{1.25434, 2.04314, 0.1}	{1.27116, 1.91, 3.99}	{{1.27121, 1.91565, 4.}, {1.27121, 1.91565, 4.}}	{1.3413, 6.51626, 3890.}	inf	2.	5.35583	{1.58459, 8.9007}	174.413	0.390399	newly	6.	13.71	{3., 25.}	147.833	0.364253	Reff	0.769437	0.779715	{0.7797, 0.77979}	1.33576	ar	38 662.	38 676.9	{38 660., 38 697.}	0.0411257
39	R0	{1.27466, 3.0741, 0.1}	{1.25553, 2.62268, 0.45}	{{1.24751, 2.5364, 0.}, {1.30094, 3.11158, 3.}}	{2.11956, 15.0504, 520.}	inf	79.	43.3292	{12.958, 71.0129}	45.7498	0.341351	newly	134.	85.64	{26., 137.}	40.5672	0.290802	Reff	0.765178	0.754141	{0.748982, 0.781232}	2.07829	ar	40 181.	40 071.8	{39 940., 40 205.}	0.321719
40	R0	{1.33045, 2.68154, 0.1}	{1.32102, 2.31502, 3.86}	{{1.31246, 2.23442, 3.}, {1.37363, 2.81014, 4.}}	{1.61742, 15.011, 3760.}	inf	10.	19.9215	{5.3811, 37.1464}	120.985	0.34009	newly	17.	40.83	{10., 76.}	159.706	0.40035	Reff	0.719255	0.714027	{0.709187, 0.742424}	1.63148	ar	45 951.	45 998.6	{45 943., 46 061.}	0.111575
41	R0	{1.37003, 2.83984, 0.1}	{1.37363, 2.81014, 3.}	{{1.37363, 2.81014, 3.}, {1.37363, 2.81014, 3.}}	{0.263126, 1.04578, 2900.}	inf	53.	39.6597	{18.8523, 62.4911}	34.1004	0.208654	newly	99.	68.93	{30., 115.}	38.2323	0.240469	Reff	0.701168	0.703196	{0.702871, 0.703458}	0.289261	ar	48 926.	48 903.1	{48 828., 49 020.}	0.134632
42	R0	{1.3003, 2.94885, 0.1}	{1.31246, 2.23442, 4.}	{{1.31246, 2.23442, 4.}, {1.31246, 2.23442, 4.}}	{0.935018, 24.2273, 3900.}	inf	49.	34.6651	{10.4579, 65.4102}	42.943	0.300558	newly	90.	75.78	{20., 137.}	44.8667	0.286137	Reff	0.726881	0.733865	{0.733462, 0.734181}	0.960901	ar	44 237.	44 179.5	{44 082., 44 287.}	0.201754
43	R0	{1.33756, 2.92653, 0.1}	{1.37363, 2.81014, 3.}	{{1.37363, 2.81014, 3.}, {1.37363, 2.81014, 3.}}	{2.69707, 3.97701, 2900.}	inf	64.	47.9801	{20.0699, 76.7441}	34.1748	0.218668	newly	97.	86.09	{35., 141.}	35.7835	0.203145	Reff	0.706272	0.72554	{0.725129, 0.725901}	2.72823	ar	47 342.	47 315.6	{47 193., 47 416.}	0.173334
44	R0	{1.35801, 2.92276, 0.1}	{1.37363, 2.81014, 3.}	{{1.37363, 2.81014, 3.}, {1.37363, 2.81014, 3.}}	{1.15057, 3.85315, 2900.}	inf	37.	37.3197	{15.6586, 57.0148}	33.6804	0.173403	newly	67.	68.01	{32., 107.}	35.0597	0.18054	Reff	0.707754	0.715893	{0.71562, 0.716179}	1.14995	ar	47 940.	47 982.	{47 903., 48 082.}	0.125866
45	R0	{1.31342, 4.02649, 0.1}	{1.29213, 3.89468, 1.01}	{{1.28596, 3.47086, 0.}, {1.31535, 3.98513, 5.}}	{1.68342, 3.57254, 1066.}	inf	207.	219.332	{175.285, 267.145}	14.6884	0.0609799	newly	308.	312.98	{246., 371.}	14.8312	0.0640634	Reff	0.755532	0.743123	{0.738962, 0.756625}	1.70324	ar	42 995.	43 097.	{42 865., 43 343.}	0.390115
46	R0	{1.37919, 3.75889, 0.1}	{1.37974, 3.3318, 5.}	{{1.37974, 3.3318, 5.}, {1.37974, 3.3318, 5.}}	{0.0398035, 11.3623, 4900.}	inf	174.	152.471	{109.511, 195.491}	18.7108	0.0934029	newly	255.	235.7	{160., 306.}	19.0588	0.0919371	Reff	0.704463	0.70504	{0.704381, 0.705599}	0.0902232	ar	49 157.	49 244.	{49 066., 49 415.}	0.271701
47	R0	{1.37458, 3.56117, 0.1}	{1.36054, 3.23048, 3.02}	{{1.36035, 3.22945, 3.}, {1.36035, 3.22945, 3.}}	{1.02873, 9.28611, 2920.}	inf	54.	62.6611	{34.2205, 91.3636}	33.023	0.1346	newly	75.	99.11	{49., 148.}	47.0533	0.175393	Reff	0.710232	0.702861	{0.702356, 0.703148}	1.0441	ar	48 478.	48 482.7	{48 366., 48 594.}	0.1452
48	R0	{1.33393, 3.78296, 0.1}	{1.31535, 3.47086, 5.}	{{1.31535, 3.47086, 5.}, {1.31535, 3.47086, 5.}}	{1.39265, 8.25018, 4900.}	inf	157.	149.877	{106.907, 188.452}	17.7985	0.0834881	newly	214.	232.5	{161., 298.}	22.3458	0.093666	Reff	0.740251	0.730036	{0.729497, 0.730595}	1.38	ar	44 885.	44 909.3	{44 700., 45 136.}	0.311708
49	R0	{1.24918, 2.39046, 0.1}	{1.27121, 1.91565, 4.}	{{1.27121, 1.91565, 4.}, {1.27121, 1.91565, 4.}}	{1.76385, 19.8628, 3900.}	inf	26.	32.4582	{8.23032, 63.8657}	63.8361	0.266747	newly	69.	87.62	{28., 185.}	67.5072	0.290679	Reff	0.801174	0.815223	{0.814816, 0.815515}	1.7536	ar	35 969.	36 028.3	{35 871., 36 222.}	0.325252
50	R0	{1.28281, 2.3032, 0.1}	{1.27204, 1.92202, 4.}	{{1.27121, 1.91565, 4.}, {1.27121, 1.91565, 4.}}	{0.932124, 16.5499, 3900.}	inf	27.	24.3913	{8.01028, 43.7992}	43.1177	0.247154	newly	59.	58.91	{14., 110.}	47.6102	0.264393	Reff	0.767787	0.761374	{0.760626, 0.761111}	0.928943	ar	40 223.	40 220.7	{40 144., 40 324.}	0.136713
